# Inaugural Charles River World Congress on Animal Models in Drug Discovery and Development

**DOI:** 10.1186/s12967-017-1274-9

**Published:** 2017-09-05

**Authors:** Jay A. Berzofsky, Lauren Gerard Koch, Steven L. Britton, Shaochen Chen, Wei Zhu, Xuanyi Ma, Anthony G. Comuzzie, Laetitia Devy-Dimanche, Ryan Feaver, Jan Grimm, Christoph Hock, Roger M. Nitsch, James B. Hoying, Aldons J. Lusis, Francesco Marincola, Josue Samayoa, Tolga Turan, David A. Pearce, Antti Nurmi, Tuulia Huhtala, Artem Shatillo, Jukka Puoliväli, Taneli Heikkinen, Timo Bragge, Kimmo Lehtimäki, Arun J. Sanyal, Kevin Strange, D. Lansing Taylor, Mark Miedel, Shanhang Jia, Alex Soto-Guterriez, Andrew Stern, Albert Gough

**Affiliations:** 1 Vaccine Branch, Center for Cancer Research, National Cancer Institute, National Institutes of Health, Bethesda, MD 20892 USA; 2 Department of Anesthesiology, University of Michigan, Ann Arbor, MI 48109 USA; 3 Department of Nano Engineering, University of California, San Diego, La Jolla, CA 92093 USA; 4 The Obesity Society, Silver Spring, MD 20910 USA; 5 EMD Serono, Inc, Billerica, MA 01821 USA; 6 HemoShear Therapeutics, LLC, Charlottesville, VA 22902 USA; 7 Neurimmune, Schlieren, Switzerland; 8 Institute for Regenerative Medicine, University of Zurich, Zurich, Switzerland; 9 Cardiovascular Innovation Institute, University of Louisville, Louisville, KY 40202 USA; 10 Angiomics, Inc., Louisville, KY 40223 USA; 11 Department of Medicine/Division of Cardiology, Department of Human Genetics, Department of Microbiology, Immunology and Molecular Genetics, University of California, Los Angeles, Los Angeles, CA 90095 USA; 12 AbbVie Immune Oncology Discovery, Redwood City, CA USA; 13 Pediatrics and Rare Diseases Group, Sanford Research, Sioux Falls, SD 57104 USA; 14 Discovery Services, Charles River, 70210 Kuopio, Finland; 15 Virginia Commonwealth University School of Medicine, Richmond, VA 23298 USA; 16 Novo Biosciences, Inc. and MDI Biological Laboratory, Bar Harbor, ME 04609 USA; 17 Drug Discovery Institute, University of Pittsburgh, Pittsburgh, PA 15261 USA; 18 Dept of Pathology, University of Pittsburgh, Pittsburgh, PA 15261 USA

## A1 Bench to bedside in oncology: translation of cancer vaccines from mouse models to human clinical trials

### Jay A. Berzofsky

#### Vaccine Branch, Center for Cancer Research, National Cancer Institute, National Institutes of Health, Bethesda, MD 20892 USA

##### **Correspondence:** Jay A. Berzofsky (berzofsj@mail.nih.gov)

*Journal of Translational Medicine* 2017, **15(Suppl 3)**: A1

We developed two cancer vaccine platforms in mouse models and translated these into human clinical trials with promising preliminary results. First, we studied TARP, a prostate cancer antigen discovered at NCI [1], mapping HLA-A2-presented epitopes, and testing these in HLA-A2-transgenic mice [2]. We then modified the amino acid sequences to improve binding to HLA-A2, and tested in mouse models whether the modified peptides were more immunogenic and induced T cells that recognized the unmodified peptide. We call this process “epitope enhancement.” We confirmed that the enhanced peptides could stimulate human T cells in vitro to kill human cancer cells expressing HLA-A2 and TARP [2]. The mouse studies led to our phase I clinical trial in patients with stage D0 prostate cancer, in which the primary tumor is removed but a rising PSA indicates microscopic recurrence, before any tumor can be seen radiographically. Patients were immunized with 2 TARP peptides either in Montanide-ISA51 or pulsed onto autologous dendritic cells (DCs). Because there was no difference in outcomes, we could pool the arms for higher statistical power. At 6 months >71% of the patients had a decreased rate of PSA rise (p = 0.0012), which has been shown to be a valid predictor of outcome. At 1 year, 74% of patients had a decreased PSA slope (p = 0.0004). By fitting to an exponential growth curve, the median tumor growth rate constant was cut in half [3]. A randomized, placebo-controlled phase II study is underway with a broader set of TARP peptides to avoid restriction to HLA-A2 patients.

Second, we developed a vaccine targeting the HER2 oncogene, responsible for about ¼ of breast cancers and a smaller % of several other cancers. For mice, we made an adenovirus expressing the extracellular and transmembrane domains of rodent HER2. In HER2-transgenic BALB/c mice that inexorably develop tumors in all 10 mammary glands, early vaccination could prevent tumor appearance [4, 5]. In wild-type BALB/c mice injected with TUBO tumor cells from the transgenic mice, the vaccine cured large (2-cm) established tumors and lung metastases [6]. The mechanism, surprisingly, was purely antibody mediated, by antibodies inhibiting HER2 phosphorylation, and was FcR independent, unlike trastuzumab. We are engaged in a clinical trial in patients with advanced metastatic HER2^+^ tumors who have failed other therapies. Among patients naïve to trastuzumab, in the second and third dose cohorts, 5/11 patients had some clinical benefit. Thus, two cancer vaccine platforms developed in transgenic mouse models were successfully translated to human trials with promising results.

**Trial registration**

NCI Trials 09-C-0139, 15-C-0075 and 15-C-0076 on TARP prostate cancer vaccine

NCI Trial 13-C-0016 on AdHER2 vaccine trial

**References**

1. Wolfgang CD, Essand M, Vincent JJ, Lee B, Pastan I. TARP: a nuclear protein expressed in prostate and breast cancer cells derived from an alternate reading frame of the T cell receptor gamma chain locus. Proc Natl Acad Sci USA. 2000;97:9437–42.
2. Oh S, Terabe M, Pendleton CD, Bhattacharyy A, Bera TK, Epel M, Reiter Y, Phillips J, Linehan WM, Kasten-Sportes C, et al. Human CTL to wild type and enhanced epitopes of a novel prostate and breast tumor-associated protein, TARP, lyse human breast cancer cells. Cancer Res 2004;64:2610–8.
3. Wood LV, Fojo A, Roberson BD, Hughes MSB, Dahut W, Gulley JL, Madan RA, Arlen PM, Sabatino M, Stroncek DF, et al. TARP vaccination is associated with slowing in PSA velocity and decreasing tumor growth rates in patients with stage D0 prostate cancer. Oncol Immunol. 2016:e1197459.
4. Sakai Y, Morrison BJ, Burke JD, Park JM, Terabe M, Janik JE, Forni G, Berzofsky JA, Morris JC. Vaccination by genetically modified dendritic cells expressing a truncated neu oncogene prevents development of breast cancer in transgenic mice. Cancer Res. 2004;64:8022–8.
5. Park JM, Terabe M, Sakai Y, Munasinghe J, Forni G, Morris JC, Berzofsky JA. Early role of CD4+ Th1 cells and antibodies in HER-2 adenovirus-vaccine protection against autochthonous mammary carcinomas. J Immunol. 2005;174:4228–36.
6. Park JM, Terabe M, Steel JC, Forni G, Sakai Y, Morris JC, Berzofsky JA. Therapy of advanced established murine breast cancer with a recombinant adenoviral ErbB-2/neu vaccine. Cancer Res. 2008;68:1979–87.

## A2 Rat models of susceptibility to complex diseases: a solution to Eroom’s Law

### Lauren Gerard Koch, Steven L. Britton

#### Department of Anesthesiology, University of Michigan, Ann Arbor, Michigan 48109 USA

##### **Correspondence:** Steven L. Britton (britton@umich.edu)

*Journal of Translational Medicine* 2017, **15(Suppl 3)**: A2

**A 30 institution consortium seeks a partner to discover effective and safe drugs utilizing rat models of multiple complex disease conditions that have a common underlying mechanism.**

**The problem**: Between 1950 and the present, the inflation-adjusted industrial development costs per drug increased nearly 100-fold to arrive at 1 billion dollars. This trend was termed Eroom’s Law (i.e., Moore’s Law backwards) by Jack Scannell. Eroom’s Law is an enigma because huge high-throughput technological gains should have raised the efficiency of research and development (R&D). A recent quantitative decision-theoretic model [1] of the R&D process led to the conclusion that Eroom’s is explained by: (1) limited creation and use of valid animal models of disease, and (2) **too much reliance on reductionist molecular approaches that are devoid of the very complexity that embodies emergent disease conditions**.

**The solution**: We foresaw this reductionist dilemma and opted for an integrative, theory-based, approach to resolve complex diseases [2]. We had noted a clinical literature that strongly linked low exercise capacity and high morbidity and mortality. By connecting this clinical observation with a theoretical base, we hypothesized that: *variation in capacity for energy transfer metabolism is the central mechanistic determinant between disease and health* (*energy transfer hypothesis: ETH*). As a predictive test of this hypothesis, we show that two-way selective breeding of genetically heterogeneous rats for low and high intrinsic treadmill running capacity (used as a surrogate for energy transfer) also produces rats that differ for disease risks. The lines are termed Low Capacity Runners (LCR) and High Capacity Runners (HCR) and after 36 generations of selection differ by over eightfold in running capacity. Consistent with the *ETH*, the LCR score high for developing numerous complex disorders (Table 1).

**Table:** Table 1 Disease/risks (LCR relative to HCR)

Metabolic syndrome	Memory and learning deficits	DNA-PK mediates disease [3]
Obesity	Spontaneous NAFLD inducible into NASH	↑ Susceptibility to infectivity
Diabetic neuropathy	Alzheimer’s neurodegeneration	↓ Metabolic flexibility
Reduced longevity	↑ Vulnerability to ventricular fibrillation	↑ Inducible cancer

Thus, the LCR and HCR contrast for low health and high health as underwritten by the common feature of capacity for energy transfer.

**Drug discovery path**: A university drug discovery consortium has been formed that is comprised of 30 institutions experienced in study of the LCR/HCR rats. This consortium seeks to couple with an industrial partner and organize to: (1) share all pre-publication outcomes in the LCR/HCR rats, (2) make suggestions for needed studies, (3) conduct industry in-house study of the rats, and (4) provide new and patented agents for testing of efficacy and safety.

**References**

1. Scannell, JW, Bosley J. When quality beats quantity: decision theory, drug discovery, and the reproducibility crisis. PLoS ONE. 2016;11(2):e0147215.
2. Koch LG, Britton SL. Theoretical and biological evaluation of the link between low exercise capacity and disease risk. Cold Spring Harb Perspect Med. 2017.
3. Park SJ, et al. DNA-PK promotes the mitochondrial, metabolic, and physical decline that occurs during aging. Cell Metab. 2017;25(5):1135–46e7.

## A3 Rapid 3D bioprinting of blood vessel network and microphysiological systems

### Shaochen Chen, Wei Zhu, Xuanyi Ma

#### Department of NanoEngineering, University of California, San Diego, La Jolla, CA 92093, USA

##### **Correspondence:** Shaochen Chen (chen168@eng.ucsd.edu)

*Journal of Translational Medicine* 2017, **15(Suppl 3)**: A3

We will present our laboratory’s recent research efforts in rapid continuous projection 3D bioprinting to create 3D scaffolds using a variety of biomaterials. These 3D biomaterials are functionalized with precise control of micro-architecture, mechanical (e.g., stiffness and Poisson’s ratio), chemical, and biological properties [1]. Design, fabrication, and experimental results will be discussed. Such functional biomaterials allow us to investigate cell-microenvironment interactions at nano- and micro-scales in response to integrated physical and chemical stimuli. From these fundamental studies we can create both in vitro and in vivo microphysiological systems such as a human liver tissue for tissue regeneration, disease modeling, and drug discovery [2].

To vascularize these engineered tissues, we have developed a prevascularization technique by using the rapid 3D bioprinting method [3]. Multiple cell types mimicking the native vascular cell composition were encapsulated directly into hydrogels with precisely controlled distribution without the need of sacrificial materials or perfusion. With regionally controlled biomaterial properties, the endothelial cells formed lumen-like structures spontaneously in vitro. In vivo implantation demonstrated the survival and progressive formation of the endothelial network in the prevascularized tissue. Anastomosis between the bioprinted endothelial network and host circulation was observed with functional blood vessels featuring red blood cells. With the superior bioprinting speed, flexibility and scalability, this new prevascularization approach can be broadly applicable to the engineering and translation of various functional tissues.

**References**

1. Ma X, Qu X, Zhu W, Li Y, Yuan S, Zhang H, Liu J, Wang P, Lai C, Zanella F, Feng G, Sheikh F, Chien S, Chen S. A deterministically patterned biomimetic human iPSC-derived hepatic model via rapid 3D bioprinting. Proceedings of the national academy of sciences (PNAS). 2016; vol 113 (no. 8), p. 2206–11.
2. Gou M, Qu X, Zhu W, Xiang M, Yang J, Zhang K, Wei Y, Chen S. Bio-inspired detoxification using 3D-printed hydrogel Nanocomposites. Nat Commun. 2014;5:3774.
3. Zhu W, Qu X, Zhu J, Ma X, Patel S, Liu J, Wang P, Lai C, Gou M, Xu Y, Zhang K, Chen S. Direct 3D bioprinting of prevascularized tissue constructs with complex microarchitecture. Biomaterials. 2017;124:106–15.

## A4 Diet preferences in large animal models of cardiovascular disease: does it matter?

### Anthony G. Comuzzie

#### The Obesity Society, Silver Spring, MD, 20910 USA

##### **Correspondence:** Anthony G. Comuzzie (tcomuzzie@obesity.org)

*Journal of Translational Medicine* 2017, **15(Suppl 3)**: A4

Dietary factors are well recognized as major contributors of risk for a variety of cardiometabolic conditions, and research in this area utilizing animal models has long taken advantage of dietary manipulation in attempting to induce the desired pathophysiological outcomes. Most of the animal-based research related to diet and risk associated with cardiovascular disease, obesity, and diabetes has, until relatively recently, focused primarily on the impact of specific dietary fats, but there is also growing interest in the impact of carbohydrates and proteins as well. While the expansion of these efforts to examine the impact of these nutritional components has expanded our knowledge of cardiometabolic disease risk and progression, the potential contribution of overall palatability on the feeding behavior of research animals has largely been ignored. Data from multiple feeding studies that we have conducted in the baboon, a large highly-omnivorous nonhuman primate species, suggests the importance of considering palatability in the development of dietary challenges to investigate the impact of nutritional components on cardiometabolic health. While all animals in a controlled research setting must consume the food which they are provided, our work indicates that their pattern of consumption, an important component in the mechanism of nutritional impact on disease risk, may vary depending on the palatability of the diet. We have found that by increasing the palatability of our challenge diets, simply by adding fruit flavoring and baking, we can obtain a pattern of consumption that is most likely more like that seen in humans, and as a result more successfully drives the development of the clinical manifestations of interest.

## A5 The translational medicine guide: asking the right question at the right time

### Laetitia Devy-Dimanche

#### EMD Serono, Inc., Billerica, MA 01821 USA

##### **Correspondence:** Laetitia Devy-Dimanche (Laetitia.Devy-Dimanche@emdserono.com)

*Journal of Translational Medicine* 2017, **15(Suppl 3)**: A5

This abstract is not included here as it has already been published [1].

**Reference**

1. Devy-Dimanche L. The translational medicine guide: asking the right question at the right time. J Pharm Drug Deliv Res. 2016;5:5.

## A6 Patient in a dish – non-alcoholic steatohepatitis (NASH) and beyond

### Ryan Feaver

#### HemoShear Therapeutics, LLC, Charlottesville, VA, 22902 USA

##### **Correspondence:** Ryan Feaver (feaver@hemoshear.com)

*Journal of Translational Medicine* 2017, **15(Suppl 3)**: A6

Non-alcoholic fatty liver disease (NAFLD) is a rapidly emerging public health crisis, affecting up to 1/3 of the U.S. population, 75% of type 2 diabetics, and 95% of obese individuals, and can progress to non-alcoholic steatohepatitis (NASH) and cirrhosis, often resulting in liver transplant or death. This is one of the most active areas in drug discovery with over 40 drugs in the preclinical and clinical phase of development, but none to date advancing beyond Phase III clinical trials or approved for therapy. The field is hampered by a lack of disease understanding and overreliance on >40 rodent models unable to map to the human response. In this presentation, we describe an in vitro approach that combines primary human hepatocytes, stellate cells, macrophages, a physiologically relevant tissue microenvironment consisting of liver sinusoid hemodynamic and transport conditions, and clinically-derived concentrations of NASH risk factors to create “NASH in a dish.” This NASH system captures critical pathophysiological drivers of NASH such as steatosis, inflammation, and fibrosis and has been validated against human NASH biopsy samples [1] and with leading clinical stage drugs. To date, we have utilized the in vitro human NASH model to survey the current drug development landscape in order to understand the therapeutic gaps, providing the principal dataset to identity new targets for NASH therapies. This “patient in a dish” concept is also being applied to identify novel targets and develop new therapies to treat pediatric rare liver diseases [2] and in the future, rare vascular diseases [3].

**References**

1. Feaver RE, Cole BK, Lawson MJ, Hoang SA, Marukian S, Blackman BR, et al. Development of an in vitro human liver system for interrogating nonalcoholic steatohepatitis. JCI Insight. 2016;1(20):e90954.
2. Chapman KA, Collado MS, Figler RA, Hoang SA, Armstrong AJ, Cui W, et al. Recapitulation of metabolic defects in a model of propionic acidemia using patient-derived primary hepatocytes. Mol Genet Metab. 2016;117(3):355–62.
3. Collado MS, Cole BK, Figler RA, Lawson M, Manka D, Simmers MB, et al. Exposure of induced pluripotent stem cell-derived vascular endothelial and smooth muscle cells in coculture to hemodynamics induces primary vascular cell-like phenotypes. Stem Cells Transl Med. 2017 Jun 19.

## A7 Development of human antibodies for the treatment of protein aggregation diseases

### Jan Grimm^1^, Christoph Hock^1,2^, Roger M Nitsch^1,2^

#### ^1^Neurimmune, Schlieren, Switzerland; ^2^Institute for Regenerative Medicine, University of Zurich, Zurich, Switzerland

##### **Correspondence:** Jan Grimm (jan.grimm@neurimmune.com)

*Journal of Translational Medicine* 2017, **15(Suppl 3)**: A7

Abnormal folding and aggregation of endogenous proteins in the brain or peripheral tissues characterizes many degenerative diseases, including Alzheimer’s and Parkinson’s disease, amyotrophic lateral sclerosis, and type-2 diabetes. The abnormal protein aggregates are stable; they can be involved in cell-to-cell propagation of pathology, and can adopt conformations with cytotoxic activities. The presence of plasma antibodies against such misfolded proteins suggests active humoral immune responses, along with the formation of B cell memory. We hypothesized that selected B cell clones triggered by neo-epitopes of pathological protein conformations encode antibodies that can block the toxicity and promote the clearance of protein aggregates. To test this hypothesis, we analyzed the memory B cell repertoires of a large cohort of healthy aged human donors, including donors with disease risk and abnormally slow progression or onset. Based on these analyses, we generated recombinant high affinity human monoclonal antibodies designed to selectively target pathological neo-epitopes within abnormal protein structures, including aggregated Aβ, tau, α-synuclein, TAR DNA-binding protein 43, superoxide dismutase 1, islet amyloid polypeptide and transthyretin. These antibodies can be effective in neutralizing toxicity, in blocking cell-to-cell propagation, and in triggering the removal of protein aggregates by microglia or macrophage-mediated phagocytosis. The results of our studies provide the scientific basis for a technology in recombinant human monoclonal antibody design, resulting in a novel class of efficacious and safe biopharmaceutical products. Aducanumab is one such human monoclonal antibody, currently in phase III clinical trials for the treatment of Alzheimer’s disease. Other candidates are in phase I for Parkinson’s disease and tauopathies, and in preclinical stage development for amyotrophic lateral sclerosis.

## A8 Microvessel-based 3D assays of angiogenesis

### James B. Hoying^1,2^

#### ^1^Cardiovascular Innovation Institute, University of Louisville, Louisville, KY 40202, USA; ^2^Angiomics, Inc. Louisville, KY 40223, USA

##### **Correspondence:** James B. Hoying (jay.hoying@louisville.edu)

*Journal of Translational Medicine* 2017, **15(Suppl 3)**: A8

Tissue health and disease involve complex interactions between the microvasculature, stroma and parenchyma of the tissue. The microvasculature itself requires a dynamic interplay between vascular and perivascular cell types to maintain proper form and function. With this in mind, we developed and implemented a versatile, enabling assay platform uniquely positioned to assess an integrated angiogenesis response in a complex environment. Involving the use of intact, isolated human microvessels in a 3D stromal environment, the assay platform recapitulates native angiogenesis for use in phenotypic screens of different neovascular behaviors. The system is based on bona fide angiogenic sprouting and neovessel growth from isolated, intact parent microvessels (e.g., arterioles, capillaries, and venules) that retain all intrinsic vascular and perivascular cells within a 3D matrix environment. This model system has been effectively used to identify and characterize putative angiogenic factors and inhibitors, evaluate microvascular instability, and define tissue dynamics during angiogenesis. Using the angiogenesis assay, we performed a phenotypic screen of a library of 128 compounds targeting a variety of epigenetic regulators (primarily histone modifications; Selleckchem, Inc.) for their effects on angiogenesis. In the screen, angiogenesis was assayed in 3D cultures of isolated microvessels under serum-free conditions that modestly promote angiogenesis, enabling us to identify agents that either stimulate or inhibit angiogenesis. In the assay, neovessels sprout from the seeded, whole parent microvessels in a regimented fashion resulting in an increase in total vessel length over time, measured as fractional microvessel area using the Cytation™ 3 scanner and Gen5 analysis software (BioTek, Inc.). This vascularizing system, based on native microvessels in a 3D environment, is a versatile assay platform, compatible with high-content analysis modalities, with proven utility in a variety of research and pharmaceutical applications. In addition, the microvessels are showing promise in regenerative medicine and tissue fabrication as a promising vascularizing solution.

## A9 Exercise capacity, aging, and longevity

### Lauren Gerard Koch, Steven L. Britton

#### Department of Anesthesiology, University of Michigan, Ann Arbor, Michigan 48109 USA

##### **Correspondence:** Lauren Gerard Koch (lgkoch@med.umich.edu)

*Journal of Translational Medicine* 2017, **15(Suppl 3)**: A9

**Translational Aspect:** Over the past few decades several large-scale epidemiological studies show that high performance on tests of maximal exercise capacity associates with lower all-cause morbidity and mortality. Concomitantly, maintaining an aerobic-based exercise program is a primary recommendation for the prevention and treatment of chronic disease, and one of the most important interventions for successful aging and longevity. Yet, there is wide inter-individual variation for exercise capacity and up to 20% of human subjects are considered “exercise resistant”; i.e., demonstrate little or no change in maximal oxygen consumption (VO2max) in response to training. The goal of our research is to develop contrasting animal model systems to systematically translate the clinical observation that variation in exercise capacity is a strong indicator of health and underlies our risk for complex disease, accelerated aging, and diminished longevity.

**Model Approach:** We used large-scale two-way artificial selection in genetically heterogeneous rats across several generations to produce two unique animal models for exploration into wide-ranging genomic-environment interactions: One model system differs in intrinsic (non-trained) capacity for maximal treadmill running—low capacity runners vs. high capacity runners, and a second model differs in capacity to respond to treadmill exercise training (acquired)—low response trainers vs. high response trainers. We maintain a breeding colony consisting of a total of ~50 families as an international research resource, retain a detailed 40-generation pedigree database, and archive biological tissues samples for molecular tracking purposes.

**Results:** We found that two-way artificial selective breeding of rats for low and high innate exercise capacity is heritable, genetically segregating, and yields rats that differ for numerous disease risks, including the metabolic syndrome, Alzheimer’s-like neurodegeneration, cognitive decline, susceptibility to inducible breast cancer, premature aging, and reduced longevity. Critically, we discovered that maximal oxygen consumption (VO2max) was a strong predictor of lifespan. Our development of rat models with similar mid-level intrinsic capacity but different magnitude for capacity to gain in response to endurance exercise training affords a unique opportunity to test whether attaining a high aerobic capacity/VO2max via training is necessary for the beneficial effects of exercise on longevity. The selection criterion for this animal model is the change in treadmill running capacity in response to 8 weeks of treadmill run training. We found that “exercise resistant” rats have low responses to training, especially for VO2max, cell remodeling, angiogenesis, mitochondrial expansion, and neurogenesis whereas the “exercise sensitive” rats display robust exercise-induced responses across lifespan, including benefit from late-life training.

**Conclusions:** Exercise intervention is regarded as the first line of defense for reducing or ameliorating several complex disease risks. Here we show that exercise genomic rat models via selection provide unbiased support that variation in exercise capacity (either intrinsic or acquired) correctly forecasts future health and disease across aging. Moreover, this original and translational animal model system represents a basic yet highly individualized underlying process that can be probed repeatedly across lifetime for more detailed information regarding efficacy and specificity within drug discovery and development.

## A10 Understanding complex interactions in metabolic and cardiovascular disease: genetics, the microbiome, sex, and diet

### Aldons J. Lusis

#### Department of Medicine/Division of Cardiology, Department of Human Genetics, Department of Microbiology, Immunology and Molecular Genetics, University of California, Los Angeles, Los Angeles, CA 90095, USA

##### **Correspondence:** Aldons J. Lusis (JLusis@mednet.ucla.edu)

*Journal of Translational Medicine* 2017, **15(Suppl 3)**: A10

One important application of animal models is, of course, to identify mechanisms through experimental perturbation. A less well-known application is to utilize natural variation to study complex interactions. Our lab studies a panel of diverse inbred strains of mice to understand interactions between genetics, sex, the microbiome, and diet in the context of cardiometabolic disease [1].

One surprising finding from our work is that genetic variation of the host is a major determinant of the composition of the gut microbiome. For example, when our panel of common inbred strains of mice were maintained in the same vivarium and fed the same diet, the heritability of gut microbiota was about 40% or higher for most genera of gut bacteria [2]. Nevertheless, the microbiome could be dramatically altered by dietary challenge (for example, switching from a chow to a high fat diet), and microbiota transplantation studies showed that the effects of the diets on the host were dependent in part upon the composition of the gut microbiota.

We also found that effects of dietary challenge were highly dependent upon genetic background, sex, and microbiome composition [3]. This is, of course, consistent with our everyday experience: we all know individuals who can eat endlessly but remain lean. While the lessons we learn of studies in mice may not be directly translatable to humans, an understanding of the basic principles will help guide human studies.

**References**

1. Orozco LD, Bennett BJ, Farber CR, Ghazalpour A, Pan A, Che N, Wen P, Qi, HX, Mutukulu A, Siemers N, Neuhaus I, Yordanova R, Gargalovic P, Pellegrini M, Kirchgessner T, Lusis AJ. Unraveling inflammatory responses using systems genetics and gene-environment interactions in macrophages. Cell. 2012;151:658–70.
2. Org E, Parks BW, Joo JW, Emert B, Schwartzman W, Kang EY, Mehrabian M, Pan C, Knight R, Gunsalus R, Drake TA, Eskin E, Lusis AJ. Genetic and environmental control of host-gut microbiota interactions. Genome Res. 2015;25:1558–69.
3. Parks BW, Sallam T, Mehrabian M, Psychogios N, Hui ST, Norheim F, Castellani LW, Rau CD, Pan C, Phun J, Zhou Z, Yang WP, Neuhaus I, Gargalovic PS, Kirchgessner TG, Graham M, Lee R, Tontonoz P, Gerszten RE, Hevener AL, Lusis AJ. Genetic architecture of insulin resistance in the mouse. Cell Metab. 2015;21:334–46.

## A11 A unifying model to explain primary and compensatory immune resilience of cancer

### Francesco Marincola, Josue Samayoa, Tolga Turan

#### AbbVie Immune Oncology Discovery, Redwood City, California, USA

##### **Correspondence:** Francesco Marincola (Francesco.marincola@abbvie.com)

*Journal of Translational Medicine* 2017, **15(Suppl 3)**: A11

Checkpoint inhibitor therapy is approximating its first horizon that encircles a landscape within which cancer cells survive immune destruction, thanks to mechanisms that simultaneously dampen its effectiveness. Beyond this horizon, a deserted landscape dominates, where immune interactions between the host and cancer cells are negligible or absent. In this desert, current immunotherapy targets are most likely irrelevant.

Available data suggest that three landscapes best portray the cancer microenvironment: an *immune*-*active*, an opposite *immune*-*deserted* and an intermediate *immune*-*depleted*. This trichotomy is observable across most solid tumors, suggesting that convergent evolutionary adaptations determine the survival and growth of cancer in the immune competent host.

We refer to the mechanisms allowing persistence of cancer in the immune-active cluster as *compensatory immune resistance* (*CIRes*). Conversely, we refer to survival of cancer in the immune-silent environment as *primary immune resistance* (*PIRes*).

To explain CIRes and PIRes, several models have been proposed that largely outnumber the fewer immune landscapes. Such discrepancy can be explained in three ways: (a) some models do not apply to human cancers, (b) there are subtler immune landscapes than those discernable by current approaches, or (c) some models describe different facets of the same phenomenology.

We previously described a transcriptional signature comprising the concordant activation of innate and adaptive immune effector mechanisms required for the occurrence of *immune*-*mediated tissue*-*specific destruction*. The latter represents a conserved mechanism determining destructive autoimmunity, clearance of pathogen-bearing cells, acute allograft rejection, graft-versus-host disease, and rejection of cancer. Thus, we termed this signature: the *Immunologic Constant of Rejection* (*ICR*) [1]. We subsequently observed that the ICR serves both as positive predictor of responsiveness to immunotherapy, and as favorable prognostic marker [2]. This observation suggests that these related phenomena represent facets within the continuum of anti-cancer immune surveillance. Such continuity suggests that signatures predictive of prolonged survival may mark *an immune*-*favorable cancer phenotype* and serve as surrogate predictors of responsiveness to anti-cancer immunotherapy when no outcome data are available [3, 4]. Based on this assumption, we built a *navigational map of cancer* to assign distinct immune-resistance models to the respective immune-landscape. The survey confirmed that immune suppression strictly goes hand-in-hand with immune activation in the favorable landscape, while the silent landscape is characterized by lack of immune modulation and by a lean oncogenic process that permits growth without activation of the host’s defense mechanisms.

**References**

1. Wang EA, Worschech, Marincola FM. The immunologic constant of rejection. Trends Immunol. 2008;29(6):256–62.
2. Galon J, et al. The continuum of cancer immunosurveillance: prognostic, predictive, and mechanistic signatures. Immunity. 2013;39(1):11–26.
3. Miller LD, et al. Immunogenic subtypes of breast cancer delineated by gene classifiers of immune responsiveness. Cancer Immunol Res. 2016;4(7):600–10.
4. Hendrickx W, et al. Identification of genetic determinants of breast cancer immune phenotypes by integrative genome-scale analysis. Oncoimmunology. 2017;6(2):e1253654.

## A12 Team science to advance therapeutics for Batten disease

### David A. Pearce

#### Pediatrics and Rare Diseases Group, Sanford Research, Sioux Falls, SD 57104, USA

##### **Correspondence:** David A. Pearce (David.pearce@sanfordhealth.org)

*Journal of Translational Medicine* 2017, **15(Suppl 3)**: A12

The fatal, primarily childhood, neurodegenerative disorders known collectively as neuronal ceroid lipofuscinoses (NCLs), are currently associated with mutations in 14 genes. The protein products of these genes (CLN1 to CLN14) differ in their function and their intracellular localization. NCL-associated proteins have been localized mostly to lysosomes (CLN1, CLN2, CLN3, CLN5, CLN7, CLN10, CLN12, and CLN13) but also the endoplasmic reticulum (CLN6 and CLN8), or in the cytosol associated to vesicular membranes (CLN4 and CLN14). Some of them, such as CLN1 (palmitoyl proteinthioesterase 1), CLN2 (tripeptidyl-peptidase 1), CLN5, CLN10 (cathepsin D), and CLN13 (cathepsin F), are soluble lysosomal proteins; others, like CLN3, CLN7, and CLN12, have been proposed to be lysosomal transmembrane proteins. Mouse models for many of these NCLs are available allowing studies of therapeutics that have led to clinical trials. The development of porcine models has more recently become important. As our understanding of the pathology of these diseases has advanced, identifying the right collaborators to execute understanding disease mechanisms, performing pre-clinical studies and moving to clinical trials has been important.

## A13 Improving translational animal models of CNS disease – the critical role of new technologies

### Antti Nurmi, Tuulia Huhtala, Artem Shatillo, Jukka Puoliväli, Taneli Heikkinen, Timo Bragge, Kimmo Lehtimäki

#### Discovery Services, Charles River, Kuopio, Finland 70210

##### **Correspondence:** Antti Nurmi (antti.nurmi@crl.com)

*Journal of Translational Medicine* 2017, **15(Suppl 3)**: A13

Animal models of central nervous system (CNS) diseases are widely used for both basic research and drug development as they provide understanding about the underlying disease pathophysiology, symptoms, and possible treatment targets that cannot be easily examined in human tissues. Despite the benefits and insights that animal models provide, the emerging view is that animal models of disease are limited in their ability to model human disease. Over the past several decades, there have been well documented failures in the clinic for drugs targeting major neurological diseases. These failures have been, at least in part, due to the lack of translational properties of animal models including methods that are used to test the efficacy of novel drugs. However, advances in new technologies to measure physiological and biochemical endpoints have armed the scientific community to more accurately and reliably understand the pathophysiology and therapeutic response of disease using new and established preclinical animal models.

In this presentation, we will go through some examples of established animal models of CNS diseases, their key properties, and examine how well they model human disease. We will also address issues related to interpretations in animal behavior studies and how valid interpretations mimic human symptoms and behaviors. We will also describe novel methods that help us build translational bridges between mice and men, in turn, improving our confidence in the preclinical data to increase translatability to the clinic. These examples include clinically applied imaging techniques, such as positron emission tomography (PET), magnetic resonance imaging (MRI), as well as more advanced animal behavioral techniques such as touch screen operant assays and kinematic motion analysis. We will also present an example of how multi-model studies in the rodent brain led to the discovery of vascular and metabolic anomalies of the liver, which has serious implications for biomedical research beyond CNS disease research. This finding highlights the importance of understanding animal models thoroughly by applying a broad selection of different types of technologies and approaches. Similar studies will improve our understanding of how well animal models represent human disease and help us find ways to improve their characteristics to accelerate preclinical CNS disease drug development.

## A14 A diet-induced animal model of nonalcoholic fatty liver disease

### Arun J. Sanyal

#### Virginia Commonwealth University School of Medicine, Richmond, VA 23298 USA

##### **Correspondence:** Arun J. Sanyal (arun.sanyal@vcuhealth.org)

*Journal of Translational Medicine* 2017, **15(Suppl 3)**: A14

Nonalcoholic fatty liver disease (NAFLD) is a common cause of chronic liver disease and a growing indication for liver transplantation for end-stage liver disease as well as liver cancer. There are currently no approved therapies for NASH. While a large number of therapeutic targets have been identified, it is not clear which targets are relevant at which stage of disease. These underscore the need for viable preclinical animal models of NASH that recapitulate all of the stages of NASH development and progression, and also recapitulate the human disease with respect to inducers, i.e., diet-induced obesity and not a specific gene knockout, insulin resistance, dyslipidemia, liver histology including the development of hepatocellular ballooning with Mallory Denk bodies, activation of pathways known to be relevant to human disease, and a transcriptomic profile similar to that in humans. We here describe such a model where the phenotype was originally noted in a B6129SF2/J (Stock# 101045) mice family and which is now shown to meet the above noted criteria in a consistent manner by brother-sister inbreeding to create an isogenic mouse strain (based on Jackson Laboratories SNP panel) which shows it to have about 60% C57 genes and 40% S129 genes. Of note, neither parent strain develops the full phenotype consistently. Chow-fed mice remain healthy and have a normal life span; however, upon high fat diet (42% calories from saturated fat, 0.1% cholesterol) with ad lib glucose fructose in amounts to mimic the general macronutrient composition seen in humans with NASH, the mice develop obesity, insulin resistance and hypercholesterolemia (including LDL-Cholesterol) and triglycerides. After 4 weeks of diet, they have profound steatosis, and by 12–14 weeks steatohepatitis which is well established by week 16 when early sinusoidal fibrosis is noted. This progresses to stage 2 fibrosis by week 24 and bridging fibrosis is seen after 36 weeks. The steatohepatitis includes classical hepatocellular ballooning with M-D bodies. At week 52, there is florid steatohepatitis and bridging with early nodule formation. Hepatic adenomas and adenocarcinomas develop in 50–60% of female and 80–90% of male mice between weeks 36–52, and most mice do not survive past week 60. There is activation of lipogenic SREBP-1c dependent genes by week 4–6 along with oxidative stress, and ER stress developing by week 16. PPAR-α activation occurs in the first 10 weeks and then declines. There is progressive inflammatory/apoptotic activation over time, and fibrogenesis from week 16 onwards. Gene enrichment analysis indicates that the pathways activated have a strong concordance with human NAFLD (FDR 0.01–0.06 at various stages). At a transcriptomic level, the gene signature of the tumors are similar to those seen in humans with NAFLD and HCC (FDR < 0.01). Proof of concept studies with pioglitazone (positive control) have been performed to demonstrate the plasticity of the model. These mice also develop progressive diastolic dysfunction and diastolic heart failure similar to those seen in obese diabetic humans and recently reported by us for NASH. Finally, this phenotype has been preserved after rederivation of the mice in two separate environments distinct from the original environment where they were derived. Together, these data indicate that this mouse captures the key elements of human disease and provide a tool to better understand the inter-relationships between pathways with disease progression, and also to test the therapeutic potential of individual or combination therapies for NAFLD, NASH, NASH with fibrosis, hepatic fibrosis or hepatocellular cancer, and even heart failure associated with obesity.

**Conflict of interest statement:** The mouse model described was validated at VCU School of Medicine but is now available through a mouse CRO Sanyal Biotechnologies of which the author is the President.

## A15 Small molecule discovery for regenerative medicine: the essential role of non-traditional animal models

### Kevin Strange

#### Novo Biosciences, Inc. and MDI Biological Laboratory, Bar Harbor, ME 04609, USA

##### **Correspondence:** Kevin Strange (kevin.strange@novobiosciences.com)

*Journal of Translational Medicine* 2017, **15(Suppl 3)**: A15

Regenerative medicine is the field of biomedical R&D and clinical practice focused on repairing, regenerating or replacing tissues and organs that have been lost or damaged due to injury, disease and the degenerative changes associated with aging. Much of regenerative medicine R&D remains focused on developing stem cell and in vitro tissue engineering therapies. Despite over 15 years of extensive research and investment, these approaches have not generated viable treatments and remain challenged by serious problems with efficacy, by their complexity and expense, and by regulatory hurdles.

Discovery and development of small molecules capable of activating innate tissue repair and regenerative processes is a newly emerging field in regenerative medicine. Small molecules have multiple advantages compared to other regenerative medicine therapeutic strategies, including greatly reduced complexity, likely lower treatment costs, reduced regulatory hurdles, ready reversibility of the therapy, and lack of ethical concerns. However, small molecule discovery and development to date has been constrained by limited understanding of the molecular mechanisms underlying regenerative processes.

Arguably, the most economical and efficient strategy for development of small molecules with regenerative medicine applications is to use “non-traditional” animal models for both target- and phenotype-based drug discovery. Countless invertebrate and lower vertebrate animals, including *Drosophila* and zebrafish, exhibit remarkable regenerative capabilities. The zebrafish in particular is able to fully regenerate many lost or damaged body parts. In contrast, humans and other mammals have limited capacity for regenerating damaged tissues, even though they possess the genetic instructions needed for building tissues and organs de novo during embryogenesis.

Novo Biosciences, Inc. and the MDI Biological Laboratory have undertaken a focused effort to develop lead small molecules capable of activating endogenous tissue regenerative mechanisms. Using genome-scale and hypothesis-driven comparative studies of organisms with both robust and limited regenerative capacity, we are identifying the regulatory gene and signaling circuits and putative drug targets that control the regeneration of lost and damaged tissues. These efforts are greatly facilitated by RegenDbase, a unique computational biology and bioinformatics tool we have developed. We also use the zebrafish for phenotype-based small molecule screening and discovery. I will discuss in detail the discovery and characterization of MSI-1436, the first and to date only small molecule capable of inducing regeneration in the adult mammalian heart following ischemic injury.

## A16 Metastatic breast cancer explored in a human, 3D, microfluidic liver chip

### D. Lansing Taylor^1^, Mark Miedel^1^, Shanhang Jia^1^, Alex Soto-Guterriez^2^, Andrew Stern^1^, Albert Gough^1^

#### ^1^University of Pittsburgh, Drug Discovery Institute, Pittsburgh, PA, 15261, USA; ^2^University of Pittsburgh, Dept of Pathology, Pittsburgh, PA, 15261, USA

##### **Correspondence:** D. Lansing Taylor (dltaylor@pitt.edu)

*Journal of Translational Medicine* 2017, **15(Suppl 3)**: A16

We have implemented quantitative systems pharmacology (QSP), an integrated and iterative computational and experimental approach to drug discovery [1], for drug discovery programs in neurodegenerative diseases, liver diseases, and metastatic cancers, including metastatic breast cancer. We have evolved a human, 3D, 4 cell type, microfluidic, liver microphysiology system (MPS) as a key experimental component of our metastatic breast cancer and liver disease programs [2, 3, 4]. The evolution of the liver MPS has been driven by the following principles: (a) recapitulate the liver acinus structure, including zonation, and function for at least one month; (b) evolve the complexity and functionality on a “fit for purpose” for efficacy testing and ADME-Tox; (c) develop and implement iPSC-derived liver cells from patients to capture heterogeneity of genomic and disease backgrounds; (d) create and implement real-time fluorescence-based biosensors [5], together with measurement of drugs, metabolites, and secreted molecules in the efflux media; (f) implement a microphysiology database to manage, analyze, and model data [6]; (g) design the liver MPS as a stand-alone liver or coupled to other organ MPS such as the intestine and kidney [7]; and (h) design should be scalable.

Recent studies have demonstrated the expression of mutations in the estrogen receptor alpha (ESR1, ER) from tumors of patients with ER+ metastatic breast cancer who had been treated with estrogen-deprivation therapy. The mutations are concentrated within the ESR1 ligand-binding domain (LBD), and their development has been demonstrated in 20–50% of ER+ metastatic breast cancer patients in anti-hormone therapy-resistant metastases.

Metastatic breast cancer involves intravasation, extravasation and “seed and soil” growth establishment in the target organ. Our initial studies are being investigated in the human liver MPS by exploring the seed and soil growth and drug sensitivity using the wild-type estrogen receptor alpha (ESR1) and two of the most common mutations, D538G and Y537S in comparison to experiments performed in 2D where we explored distinguishable phenotypes that these mutations may confer using genome-editing methods [8]. We utilized a luciferase-based assay and an endogenous phospho-ER immunoblot analysis to characterize the estrogen response of ER mutants. Expression of either mutant conferred estrogen-independent ER transactivation and ser (118) phosphorylation. While ER transactivation and phosphorylation in Y537S-expressing clones showed no estrogen dependence, D538G mutants demonstrated an enhanced estrogen-dependent response in both assays. Additionally, both mutations conferred significant resistance to ER antagonists with Y537S mutants displaying greater resistance than D538G mutants [8]. The same studies are in progress within the liver MPS.

**References**

1. Stern AM, Schurdak, ME, Bahar, I, Berg, JM, DL Taylor. A perspective on implementing a quantitative systems pharmacology platform for drug discovery and the advancement of personalized medicine. J Biomol Screen. 2016;21:521–34.
2. Vernetti L, Senutovitch N, Boltz R, DeBiasio R, Shun TY, Gough A, Taylor DL. A human liver microphysiology platform for investigating physiology, drug safety and disease models. Exp Biol Med. 2015;241:101–14.
3. Lee-Montiel F, George S, Gough A, Vernetti LA and Taylor DL. Computational and experimental demonstration of oxygen tensions supporting zonation in a human liver microphysiology system. Exp Biol and Med. 2017; Published on line: https://doi.org/10.1177/1535370217703978.
4. Clark A, Ma B, Taylor DL, Griffith L, Wells A. Liver metastases: microenvironments and ex vivo models. Exp Biol Med Liver metastases: microenvironments and ex vivo models. 2016; Exp Biol Med. 241(15):1639–52.
5. Senutovitch N, Vernetti, L, Boltz, R, DeBiasio, R, Gough, A, Taylor, DL. Fluorescent protein biosensors applied to microphysiological systems. Exp Biol Med. 2015;240:795–808.
6. Gough A, Vernetti L, Bergenthal L, Shun T-Y, Taylor DL. The microphysiology database for analyzing and modeling compound interactions with human and animal organ models. Appl In Vitro Tox 2. 2016;(2):103–17.
7. Vernetti L, Gough A, Baetz N, Blutt S, Broughman JR, Brown JA, Foulke-Abel J, Hasan N, In J, Kelly E, Kovbasnjuk O, Repper J, Senutovitch N, Stabb J, Yeung C, Zachos NC, Donowitz M, Estes M, Himmelfarb J, Truskey G, Wikswo JP, Taylor DL. Functional coupling of human microphysiology systems: intestine, liver, kidney proximal tubule, blood–brain barrier and skeletal muscle. Sci Rep. 2017;7:42296.
8. Shanhang J^*^, Miedel MT^*^, Ngo M, Hessenius R, Wang P, Bahreini A, Li Z, Ding Z, Chen N Shun TY, Zuckerman DM, Taylor DL, Puhalla SL, Lee AV, Oesterreich S, Stern AM. Clinically observed estrogen receptor alpha mutations within the ligand-binding domain confer distinguishable phenotypes indicative of Darwinian-like somatic evolution. PLoS ONE. 2017; (manuscript in review). [* joint first author].

